# Examination of the Relationship between Peripheral Inflammation Markers and Impulsivity and Aggression in Schizophrenia Patients Involved and Not Involved in Crime

**DOI:** 10.3390/jpm13030475

**Published:** 2023-03-06

**Authors:** Suheda Kaya, Gülay Taşcı, Nülüfer Kılıç, Hüsna Karadayı, Filiz Özsoy, Murad Atmaca

**Affiliations:** 1 Elazıg Mental Health Hospital, Elazığ 23100, Turkey; 2 Elazığ Fethi Sekin City Hospital, Elazığ 23100, Turkey; 3 Department of Psychiatry, School of Medicine, Tokat Gaziosmanpaşa University, Tokat 60100, Turkey; 4 Department of Psychiatry, School of Medicine, Fırat University, Elazığ 23100, Turkey

**Keywords:** aggression, crime, CRP, inflammation, schizophrenia, violence

## Abstract

Aim: The aim of this study was to examine the relationship between peripheral inflammatory markers and aggression and impulsivity in schizophrenia patients with and without criminal histories. Materials and Methods: The study was conducted with patients with schizophrenia involved in crimes and hospitalized in the Forensic Psychiatry ward of Elazığ Fethi Sekin City Hospital and patients with schizophrenia not involved in crimes and hospitalized in the psychiatry ward of Elazığ Mental Health and Diseases Hospital. All participants completed the Buss–Waren Aggression Scale (BWAS), the Barratt Impulsiveness Scale-11 (BIS-11), and the Positive and Negative Symptom Scale (PANSS). Before treatment, venous blood samples were taken for laboratory measurements on the first day of hospitalization. Results: All participants were male. The mean age of those involved in a crime was 39 ± 9.7 years, while the mean age of those not involved in a crime was 41.2 ± 10.7 years. The PANSS all subscale and total scores of the patients with schizophrenia who were involved in a crime were significantly higher than the group who were not involved (*p* values were *p* < 0.001, *p* = 0.001, *p* = 0.043, *p* = 0.001, respectively). The BWAS—physical aggression (*p* = 0.007) and total scores of the scale (*p* = 0.046) and BIS-11—inability to plan (*p* = 0.002) scores of the group involved in a crime were higher than the group not involved. As for laboratory parameters, MCH, MCHC, PDW, eosinophils, basophils, RDW-CV, and RDW-SD values were significantly higher in those involved in crime, while MPV, creatinine, albumin, and LDH values were lower. CRP and CRP/albumin values were significantly higher, while neutrophil/albumin values were significantly lower in those who committed murder in the first degree than those who committed other crimes. Conclusion: Based on our results, we found that inflammatory agents were significantly increased in forensic schizophrenia patients with high aggression scores. Significant correlations between some inflammatory factors and impulsivity and aggression scores and differences in these factors according to crime types showed that these factors might be related to violence and criminal behavior. There is a need for further large-scale studies on this subject at different stages of the disease.

## 1. Introduction

Schizophrenia is a chronic neurodevelopmental disorder that begins in childhood [1]. Many psychiatric disorders, particularly schizophrenia, have been linked to increased impulsivity and aggression [2]. Aggression in schizophrenia has been linked to factors such as psychotic symptoms, psychopathy level, impulsivity, alcohol and substance use, and irregular use of medication [3]. It has been emphasized that the impulsivity seen in schizophrenia causes aggression and that this is due to a malfunction of the frontotemporal circuit in the brain [4]. Many studies have found that patients with schizophrenia have higher rates of crime and aggression than the general population [4,5]. Individuals with major psychiatric disorders, the majority of whom are schizophrenia patients, commit approximately 5% of serious violent crimes [6]. According to Soyka et al., 10.7% of 1662 schizophrenia patients hospitalized in Germany committed a crime [5]. After it was discovered that the frequency of committing the crime was higher in patients with schizophrenia, researchers began to investigate risk factors that are effective in committing a crime [6,7].

Many studies have shown that inflammation plays an important role in the etiology of schizophrenia [8,9,10]. It has been reported that proinflammatory cytokines decrease the monoamine level in the brain, increase the neuroendocrine response, and disrupt the plasticity of the brain. Early-life stress has also been identified as a factor that contributes to neuroinflammation. Inflammation has been linked to pathophysiology and clinical status [11]. A small number of studies have found that clinical conditions such as aggression and impulsivity may be linked to inflammatory factors [12,13]. It has been shown that there is a significant relationship between inflammatory factors and committal in diseases such as antisocial personality disorder and bipolar affective disorder, where aggression and impulsivity are prominent [13,14]. Another study compared schizophrenia patients who committed murder in the first degree to those who committed other crimes, and it was found that some peripheral inflammatory factors were higher in those who committed intentional homicide [15]. However, no study was found in the literature that examined the relationship between peripheral inflammatory markers, aggression, impulsivity, and criminal behavior in schizophrenia patients. Based on this information, it was hypothesized that impulsivity and aggression scores would be increased in schizophrenia patients involved in criminal activity. Second, it was hypothesized that inflammation markers would be higher in criminal patients. Based on these hypotheses, this study aimed to examine the relationship between peripheral inflammatory factors and aggression and impulsivity in schizophrenia patients with and without criminal histories.

## 2. Materials and Methods

### 2.1. Ethics Committee Approval

The study was approved by Fırat University Non-Interventional Local Ethics Committee with project number 2022/03-33 (Meeting date: 24 February 2022). The study was conducted in accordance with the Declaration of Helsinki. 

### 2.2. Criteria for Inclusion and Exclusion

The study was planned prospectively. The study included 57 schizophrenia patients involved in a crime and hospitalized in Elazığ Fethi Sekin City Hospital’s forensic psychiatry ward and 56 schizophrenia patients who met the study criteria and were hospitalized in Elazığ Mental Health and Diseases Hospital’s psychiatry ward. The study included patients between the ages of 18 and 65 who were diagnosed with schizophrenia using DSM-5 criteria. The study excluded illiterate patients, patients with mental retardation, patients with alcohol/substance use disorders, and patients with personality disorders. The study excluded patients with poor general health, chronic diseases requiring medical treatment, renal and hepatic dysfunction, known malignancy, and local and/or systemic inflammatory diseases. 

All participants who volunteered to participate in the study provided verbal and written informed consent forms. The psychiatrist who conducted the evaluation then administered a sociodemographic and clinical data form, the Buss–Waren Aggression Scale (BWAS), the Barratt Impulsiveness Scale-11 (BIS-11), and the Positive and Negative Symptom Scale (PANSS). Venous blood samples were collected on an empty stomach on the day of admission to the ward before the start of psychiatric treatment. 

### 2.3. Data Collection Tools 

The demographic and clinical assessment form includes demographic information such as age, marital status, and level of education. Furthermore, this form includes clinical evaluation questions such as the participants’ previous judicial problems, whether or not they have been imprisoned, and the offense for which they were imprisoned. 

The Buss–Waren Aggression Scale (BWAS). In 2000, Buss and Warren [16] updated the scale developed by Buss and Perry [17] to assess anger and aggression. It has a Likert scale, a five-point scale, and 34 items. The scale is divided into five subscales: physical aggression, verbal aggression, anger, hostility, and indirect aggression. Demirtaş Madran conducted the Turkish validity and reliability study [18].

The Barratt Impulsiveness Scale-11 (BIS-11) is used to determine impulsivity. It has three subscales: attention (inattention and cognitive disorganization), motor (motor impulsivity and impatience), and inability to plan (inability to maintain control and intolerance of cognitive confusion). In addition to these subscales, the total score of the scale is computed, and the higher the total BIS-11 score, the higher the person’s impulsivity level. Güleç et al. conducted the BIS-11 validity and reliability study in Türkiye [19,20].

The Positive and Negative Symptom Scale (PANSS) was developed by Kay et al. (1987), and it is a 30-item, semi-structured interview scale with a 7-point severity rating. Of these 30 items, 18 were adapted from the Brief Psychiatric Rating Scale (BPRS) and 12 from the Psychopathology Rating Scale. Seven items are from the positive syndrome subscale, seven from the negative syndrome subscale, and the remaining sixteen are from the general psychopathology subscale. Kostakoğlu et al. (1999) conducted a Turkish reliability and validity study of the scale [21,22]. 

### 2.4. Laboratory Samples

Blood was collected from the antecubital vein of all participants after an average of 12 h of fasting and stored in EDTA tubes. A Beckman Coulter LH 750 (impedance method) analyzer was used for a complete blood cell count. Albumin level was determined with a spectrophotometer (Cobas 8000 series c702 modular analyzer). CRP level (Wako Chemicals, Osaka, Japan) was determined on a Hitachi 7600 chemistry analyzer (Hitachi, Tokyo, Japan). White blood cells, hemoglobin, hematocrit, platelets, mean platelet volume (MPV), Platelet Distribution Width (PDW), and Percent blood thrombus (PCT), leukocytes (neutrophils, lymphocytes, eosinophils, monocytes, basophils), red blood cell distribution width (RDW) were recorded. Neutrophil/lymphocyte ratio (NLO), platelet/lymphocyte ratio (PLO), monocyte/lymphocyte (MLO), CRP/albumin (CAR), and neutrophil/albumin (NAR) values were then calculated manually from the blood results. 

### 2.5. Statistical Analysis

The statistical package for social sciences (SPSS; SPSS Inc., Chicago, IL, USA) 22 package program was used to assess the analyses. For discrete data, descriptive information was shown as n,% values, and for continuous data, mean, standard deviation, and median interquartile range (values between the 25th and 75th percentiles). The Kolmogorov–Smirnov test was used to determine whether continuous factors were in compliance with the normal distribution. Student’s *t*-test was used for normally distributed variables, and the Mann–Whitney U-test was used for non-normally distributed variables of paired group analyses. To evaluate categorical variables between groups, Pearson’s chi-square analysis was used. Pearson’s correlation test was used for those with normal distribution, and Spearman’s correlation test was used for those who did not fit the normal distribution to examine the relationship between continuous variables. A binary logistic regression analysis was used to forecast who would commit crimes. The Enter method was used to create the model, which also included the significant findings from the earlier tests. The analysis accepted *p* < 0.05 as the statistical significance threshold.

## 3. Results

### 3.1. Demographic Characteristics of the Participants 

For this study, 70 people in the schizophrenia patient group who committed crimes and 73 people in the patient group who did not commit crimes were interviewed. A total of 113 patients who met the inclusion criteria were included in our study. All of the participants were male, and the mean age of those who were involved in a crime was 39 ± 9.7 years, while the mean age of those who were not involved in a crime was 41.2 ± 10.7 years (*p* = 0.254). While 57.9% of those who were involved in a crime were single, 76.8% of those who were not involved in a crime were single. There was a significant difference between the groups in terms of marital status (*p* = 0.041). The rate of patients whose parents lived together was 47.4% and was lower than the other group (*p* < 0.001). The rate of substance use (12.3%) in the group involved in a crime was significantly higher than the group not involved in a crime (0%) (*p* = 0.013). The rate of tattoo/tattooing in the patients who were involved in a crime was also higher than in patients who were not involved in a crime (*p* = 0.032). Demographic data and clinical characteristics of the participants are given in Table 1. 

### 3.2. The Comparison of Scale Scores of Groups

All subscale and total PANSS scores of schizophrenia patients who were involved in a crime were significantly higher than the non-crime group. PANSS—positive (*p* < 0.001), PANSS—negative (*p* = 0.001), PANSS—general psychopathology (*p* = 0.043), and PANSS—total (*p* = 0.001) scores were higher in the crime-involved group. The BWAS physical aggression (*p* = 0.007) and total scores of the scale (*p* = 0.046), and BIS-11 inability to plan (*p* = 0.002) scores of the group involved in a crime were higher than the group not involved. The scale scores of the participants are given in Table 2. 

### 3.3. The Results of Participants’ Laboratory Parameters

No significant difference was found between the NLO, MLO, and PLO values of the patient groups with and without a criminal history. However, MCH (*p* = 0.004), MCHC (*p* < 0.001), PDW (*p* = 0.023), eosinophil (*p* = 0.006), basophil (*p* = 0.021), RDW-CV (*p* < 0.001), and RDW-SD (*p* = 0.002) values of the group involved in a crime were significantly higher than the group not involved in a crime. MPV (*p* < 0.001), creatinine (*p* < 0.001), albumin (*p* = 0.001), and LDH (*p* < 0.001) values were lower in patients who were involved in a crime (Table 3).

### 3.4. Results of Correlation Analysis

When the correlation between the scales applied to the patients and laboratory parameters was examined, it was found that there was a significant positive correlation between BWAS physical aggression and MCV, RDW-SD, and a significant negative correlation between MPV and PCT. BWAS verbal aggression and HGB and MCV had a significant positive correlation. BWAS anger had a significant positive correlation with MCV and MCH and a significant negative correlation with PCT, LYM, and total protein. There is a significant negative relationship between BWAS indirect and PCT. The BWAS total score and PCT had a significant negative correlation. BIS-11 attentional impulsiveness and CT had a significant negative correlation. The BIS-11 motor had a significant positive correlation with MCV, MCH, and RDW-SD. BIS-11 inability to plan had a significant positive correlation with RDW-CV and RDW-SD and a significant negative correlation with WBC, PCT, NEU, LYM, total protein, and albumin. BIS-11 total had a significant positive correlation with MCV, MCH, and RDW-SD and a significant negative correlation with WBC, MPV, LYM, and MON (Table 4).

### 3.5. Logistic Regression Analysis Results

According to the logistic regression analysis, those whose parents were not together and those who had previously been imprisoned had a higher risk of involvement in a crime. Furthermore, MCH, RDW-CV, and RDW-SD values were discovered to predict criminal involvement (Table 5).

In addition, when laboratory parameters were analyzed according to crime type, those who committed murder in the first degree had significantly higher CRP (*p* = 0.045) and CRP/albumin (*p* = 0.034) values than those who committed other crimes. Those who committed murder in the first degree had significantly lower neutrophil/albumin levels than those who committed other crimes (*p* = 0.029).

## 4. Discussion

MCH, MCHC, PDW, eosinophil, basophil, RDW-CV, and RDW-SD values were found to be significantly higher in those involved in a crime compared to those not involved in our study on the relationship between peripheral inflammation markers and impulsivity and aggression in schizophrenia patients with and without involvement in a crime. MPV, creatinine, albumin, and LDH levels were significantly lower in the criminal group. All PANSS subscales and total scores were found to be significantly higher in patients who had been involved in a crime than in patients who had not been involved in a crime. The BWAS physical aggression and total aggression scores, as well as the BIS-11 inability to plan scores, were found to be higher in patients with schizophrenia who were involved in a crime than in patients who were not involved in a crime. Aggression had a positive correlation with MCV, MCH, RDW-SD, and HGB values and a negative correlation with MPV, PCT, LYM, and total protein values. There was a positive correlation between impulsivity and MCV, MCH, RDW-SD, and RDW-CV values and a negative correlation between triglycerides, WBC, MPV, PCT, NEU, MON, LYM, total protein, and albumin. CRP and CRP/albumin values were significantly higher, while neutrophil/albumin values were significantly lower in those who committed murder in the first degree than those who committed other crimes.

The link between crime, violence, homicidal behavior and inflammation in mental disorders has only recently begun to be studied [8,12,13,14,15,23]. However, no research has been conducted to investigate the relationship between peripheral inflammation markers and impulsivity and aggression in schizophrenia patients with and without criminal involvement. We found no difference in NLO, PLO, and MLO values between patients who were involved in crime and those who were not in our study, which is the first of its kind in the literature. Similar to our results, the NLO, MLO, and PLO values of patients involved in a crime and those who were not involved in a crime were not found to be different in a study of bipolar disorder patients. These values, however, were calculated to be lower in patients when compared to healthy controls [14]. A modified open aggression scale was used to divide patients with schizophrenia into two groups with and without aggressive behaviors in a study on schizophrenia patients. The NLO value was found to be higher in schizophrenia patients with aggressive behavior than in the other group in this study. NLO was found to be a clear biomarker for aggression in schizophrenia patients in the same study [24]. 

MCH, MCHC, PDW, eosinophils, basophils, RDW-CV, and RDW-SD values, which have been found in a few studies, were found to be increased in schizophrenia patients involved in crime in our study. The MPV value was found to be low. The values of MPV, PDW, and PCT indicate the activity and reactivity of platelet functions. MPV and PDW values are inversely proportional, and MPV is considered a more specific marker than PDW [25]. Monoamine oxidase (MAO) activity on platelets affects the serotonergic and dopaminergic systems [26]. It is also accepted that the changes occurring in the mitochondria of platelets are a peripheral model for neurons [27]. It has been shown that dysregulation/disruption in platelet activity may be involved in the pathophysiology of psychiatric disorders using these mechanisms [26,27]. A link has been established in the literature between aggression, suicide, and homicidal behavior and a decline in platelet activity [13,28,29]. In a study on patients with self-destruction attempts, the MPV value was found to be high in violent self-mutilation attempts [28]. MPV values were found to be low and PDW values were found to be high in a study of bipolar disorder patients who were/were not involved in crime [14]. Similarly to this study, we found that MPV values were low and PDW values were high in schizophrenia patients involved in crime. Because the number of studies conducted with criminally involved patients in the literature is quite limited [14,15], it is not possible to generalize the results obtained. However, it is expected that these data, which are compatible with one another, will contribute to the literature. Few studies have included MCH, MCHC, eosinophil, basophil, RDW-CV, and RDW-SD values. All values were not analyzed together in these limited studies [14,28,29]. Patients with bipolar disorder who were involved in a crime had lower eosinophil, RDW-CV, and RDW-SD values than those who were not involved in a crime. This study did not include MCH and MCHC values [14]. Eosinophil and basophil levels were found to be low in a study of patients who attempted self-destruction. MCH, MCHC, RDW-CV, and RDW-SD values were not examined in this study [29]. MCH and MCHC values were found to be higher in violent self-destructions than in non-violent ones. RDW-CV values did not differ between groups in the same study [28]. Inflammatory markers such as MCH, MCHC, eosinophils, basophils, RDW-CV, and RDW-SD, which have been studied in a small number of studies and obtained different results, need to be investigated further. 

Creatinine, albumin, and LDH levels, which we examined last among laboratory parameters, were found to be low in patients involved in a crime. CRP and CRP/albumin (CAR) levels were not different between groups. CRP and CAR were found to be higher in the group with more aggression in a study that looked at the relationship between impulsivity, aggression, and inflammation in schizophrenia patients. In this study, it was found that as patients’ aggression increased, albumin levels decreased and CRP levels increased [15]. Although CRP and CAR levels did not differ between groups, impulsivity and albumin levels were both negatively correlated. CRP and CAR levels were higher in schizophrenia patients who committed murder in the first degree than in patients who committed other crimes. When the patients were analyzed according to the type of crime, although there was no difference between the neutrophil values, the neutrophil/albumin value was lower in those who committed murder in the first degree because the albumin value decreased. Since our study was the first study to examine these parameters in the literature, these values obtained are quite valuable, but it is difficult to generalize. However, as a common result, the inverse relationship between impulsivity, aggression, and albumin should be kept in mind in the evaluation of patients. 

When the scales applied to the patients were evaluated, we found that all subscales and total scores of the PANSS scale were significantly higher in the crime-involved group than in the non-crime-involved group. A study in this field found no difference in the BWAS and PANSS scores of schizophrenia patients with and without criminal involvement. However, patients who committed recurrent crimes had higher BWAS physical aggression and total aggression scores than those who committed one-time crimes [30]. According to our findings, patients who were involved in a crime had higher BWAS physical aggression and total aggression scores, as well as higher BIS-11 inability to plan scores, than those who were not. According to some studies, schizophrenia patients who were involved in a crime had a low educational level and a high rate of divorce [30,31]. Moreover, smoking and substance abuse were found to be more prevalent in the group involved in a crime [30]. While smoking and alcohol use did not differ between groups, substance use was higher in the group involved in a crime. Furthermore, we found that some inflammatory parameters were positively and some were negatively associated with aggression in our findings. Furthermore, the group involved in the crime included patients whose parents had divorced and who had previously served time in prison. This result was consistent with the literature. According to studies, schizophrenia patients who are involved in a crime have separated parents and a criminal/prison history [30,31]. There are many studies with artificial intelligence in the literature for the detection, diagnosis and classification of diseases [32,33,34,35,36,37,38].

## 5. Limitations and Strengths

The first limitation is that all of the participants were men. Another limitation is the relatively small number of participants. Another limitation is the lack of a healthy control group, as well as the failure to question the groups’ dietary and exercise habits. Finally, while smoking did not result in a statistical difference, data such as the year and type of cigarettes smoked were not examined. Although they cannot access substances such as methamphetamine because substance use is prohibited in prison, we can say that inflammatory factors may have increased in those with a history of substance use. This is one of the confounding factors. These limitations restrict the generalizability of data obtained. More research should be conducted on conditions that affect inflammatory processes, such as substance-induced psychosis, at various stages of the disease, in larger sample groups, and in groups with similar gender distribution.

## 6. Conclusions

In conclusion, based on our results, we found that inflammatory agents were significantly increased in forensic schizophrenia patients with high aggression scores. Furthermore, some inflammatory levels were positively and negatively correlated with the patients’ impulsivity and aggression scores. There were significant differences in markers of inflammation by type of crime. One of the important results of our study is that CRP and CAR values are especially high in those who commit murder. However, given the nature of this serious mental illness, using these markers to predict violent activity is not entirely appropriate. However, the significant correlations between some inflammatory factors and impulsivity and aggression scores showed that these factors might be associated with violence and criminal behavior. Studies with a larger number of patients, including different stages of the disease, will be important in predicting criminal behavior in mental diseases. 

## Figures and Tables

**Table 1:** Demographic data and clinical characteristics of the participants.

		Group of Patients Involved in Crime		Non-Criminal Patient Group		*p*
		(n)	%	(n)	%	
Age (Mean ± SD) *		39.0 ± 9.7		41.2 ± 10.7		0.254
Marital Status	Single	33	57.9	43	76.8	**0.041**
	Married	15	26.3	11	19.6	
	Widowed/Divorced/Separated	9	15.8	2	3.6	
Educational Status	Middle School And Below	38	66.7	37	66.1	0.947
	High School And Above	19	33.3	19	33.9	
Social Security	There İs	52	91.2	43	76.8	**0.036**
	No	5	8.8	13	23.2	
Mother is Alive	Yes	40	70.2	40	71.4	0.884
	No	17	29.8	16	28.6	
Father İs Alive	Yes	27	47.4	27	48.2	0.928
	No	30	52.6	29	51.8	
Are The Parents Together?	Yes	27	47.4	54	96.4	**<0.001**
	No	30	52.6	2	3.6	
Has He Been İn Prison Before?	Yes	33	57.9	3	5.4	**<0.001**
	No	24	42.1	53	94.6	
Cigarette	Yes	43	75.4	41	73.2	0.787
	No	14	24.6	15	26.8	
Alcohol	Yes	8	14.0	4	7.1	0.234
	No	49	86.0	52	92.9	
Matter	Yes	7	12.3	0	0	**0.013**
	No	50	87.7	56	100.0	
Previous İnpatient Treatment	Yes	43	75.4	49	87.5	0.099
	No	14	24.6	7	12.5	
Additional Medical İllness	Yes	3	5.3	0	0	0.243
	No	54	94.7	56	100.0	
Psychiatric Treatment İn The Family	Yes	10	17.5	14	25.0	0.333
	No	47	82.5	42	75.0	
Incision/Razor/Face Scar On His Body	Yes	14	24.6	9	16.1	0.262
	No	43	75.4	47	83.9	
Suicide Attempt	Yes	14	24.6	10	17.9	0.384
	No	43	75.4	46	82.1	
Tattoo	Yes	8	14.0	1	1.8	**0.032**
	No	49	86.0	55	98.2	

* Student’s *t*-test was used in the examination for the age column, while chi-square test was used for the other columns. The age column is represented as a mean ± standard deviation, while the other data are represented as N (%). The numbers in bold are *p* < 0.05.

**Table 2:** The comparison of scale scores of groups.

	Group of Patients Involved in Crime	Non-Criminal Patient Group	*p*
	Mean ± SD	Mean ± SD	
PANSS—positive	23.2 ± 11.7	15.3 ± 8.6	**<0001**
PANSS—negative	24.8 ± 11.3	18.2 ± 9.1	**0.001**
PANSS—general level of functionality	44.6 ± 16.0	38.5 ± 15.8	**0.043**
PANNS—total score	92.9 ± 33.2	71.8 ± 30.1	**0.001**
BWAS—physical aggression	20.2 ± 10.2	15.9 ± 6.3	**0.007**
BWAS—verbal aggression	13.7 ± 5.3	12.2 ± 3.9	0.084
BWAS—anger	17.7 ± 7.8	15.4 ± 5.7	0.069
BWAS—enmity	19.9 ± 9.9	19.3 ± 5.8	0.710
BWAS—total score	85.4 ± 37.3	73.9 ± 20.3	**0.046**
BIS-11—caution	17.7 ± 7.0	16.6 ± 6.7	0.286
BIS-11—not making plans	31.0 ± 7.6	26.7 ± 6.5	**0.002**
	Mann–Whitney U test results		
BWAS—indirect aggression	11.0 (8.0–18.5)	10.0 (9.0–13.0)	0.198
BIS-11—engine	20.0 (16.0–28.0)	19.5 (16.0–24.0)	0.085
BIS-11—total score	69.0 (57.0–75.0)	64.5 (54.0–70.5)	0.062

Values given in the Table. The mean ± standard deviation is shown in the upper part of the Table, and the median interquartile range is shown in the lower part. In the calculations, the Student’s *t*-test was applied to the upper part of the Table, while the Mann–Whitney U test was applied to the lower part. At *p* < 0.05, values in bold are considered statistically significant.

**Table 3:** Blood value comparisons of the groups.

	Group of Patients Involved in Crime	Non-Criminal Patient Group	*p*
	Mean ± SD	Mean ± SD	
WBC	8.8 ± 3.7	8.8 ± 2.9	0.932
RBC	5.0 ± 0.5	5.1 ± 0.5	0.245
HGB	15.2 ± 1.5	15.0 ± 1.5	0.399
MCV	89.4 ± 5.3	88.2 ± 5.6	0.273
MCH	30.4 ± 2.2	29.3 ± 2.0	**0.004**
MCHC	33.9 ± 0.8	33.2 ± 1.1	**<0.001**
PLT	264.3 ± 60.3	256.4 ± 59.9	0.487
MPV	8.3 ± 0.9	9.7 ± 1.0	**<0.001**
PCT	0.22 ± 0.05	0.42 ± 1.33	0.237
PDW	16.6 ± 1.0	12.5 ± 13.0	**0.023**
NEU	5.4 ± 1.8	5.7 ± 2.4	0.552
LYM	2.3 ± 2.4	2.3 ± 1.5	0.943
MON	0.72 ± 0.25	0.70 ± 0.54	0.889
RDW-CV	14.0 ± 1.3	12.7 ± 0.8	**<0.001**
RDW-SD	43.6 ± 3.8	41.4 ± 3.5	**0.002**
Glucose	97.8 ± 20.9	107.9 ± 51.4	0.176
Urea	28.6 ± 11.0	28.7 ± 10.4	0.963
Creatinine	0.84 ± 0.12	0.99 ± 0.14	**<0.001**
Cholesterol	181.0 ± 36.8	181.0 ± 42.4	1.000
HDL	41.4 ± 8.1	42.9 ± 11.6	0.446
LDL	109.6 ± 32.1	110.3 ± 34.1	0.904
TG	153.8 ± 89.9	145.5 ± 124.9	0.694
Nötrofil/albümin	0.13 ± 0.04	0.13 ± 0.05	0.887
	Mann–Whitney U Test Results		
	Median (IQR) Values		
HCT	44.9 (41.4–48.0)	45.4 (41.7–47.9)	0.552
EOS	0.15 (0.10–0.28)	0.10 (0.02–0.18)	**0.006**
BAS	0.07 (0.00–0.10)	0.03 (0.02–0.04)	**0.021**
Albumin	43.0 (40.0–44.0)	45.0 (42.0–47.0)	**0.001**
AST	22.0 (18.0–27.0)	24.0 (16.5–32.0)	0.443
ALT	20.0 (15.0–27.0)	17.5 (13.0–25.0)	0.224
LDH	215.0 (199.0–251.0)	305.5 (257.5–356.0)	**<0.001**
CRP	3.7 (1.9–13.4)	3.5 (2.3–6.6)	0.561
CRP/albümin	0.09 (0.05–0.31)	0.08 (0.05–0.15)	0.422
Nötrofil/lenfosit	2.6 (2.0–3.8)	2.6 (1.8–4.0)	0.845
Monosit/lenfosit	0.38 (0.26–0.49)	0.29 (0.25–0.41)	0.153
Platelet/lenfosit	136.8 (99.2–186.4)	118.2 (93.6–169.3)	0.318

The values in the Table are the results of the Student’s *t*-test in the upper columns and the Mann–Whitney U test in the lower columns. The numbers in bold are *p* < 0.05.

**Table 4:** Results of correlation analysis.

	BWAS						BIS-11			
	1	2	3	4	5	6	1	2	3	4
Wbc	−0.143	−0.104	−0.154	−0.136	−0.174	−0.145	−0.184	−0.148	**−0.292**	**−0.241**
Hgb	0.028	**0.194**	−0.047	0.103	0.010	0.062	0.028	0.073	−0.093	−0.013
Mcv	**0.207**	**0.203**	**0.222**	**0.117**	−0.070	0.154	0.127	**0.265**	0.166	0.241
Mch	0.176	0.183	**0.189**	0.070	−0.021	0.124	0.160	**0.280**	0.168	**0.257**
Mchc	−0.034	−0.003	−0.008	−0.071	0.068	−0.022	0.037	0.042	0.030	0.057
Mpv	**−0.205**	−0.092	−0.094	−0.002	−0.158	−0.139	−0.139	−0.165	−0.173	**−0.200**
Pct	**−0.200**	−0.106	**−0.187**	−0.108	**−0.243**	**−0.199**	−0.153	−0.118	**−0.244**	−0.175
Pdw	0.090	0.090	0.077	−0.033	0.030	0.036	−0.099	0.051	0.187	0.066
Neu	−0.106	−0.093	−0.095	−0.082	−0.159	−0.108	−0.128	−0.130	**−0.226**	−0.187
Lym	−0.126	−0.147	**−0.190**	−0.180	−0.125	−0.160	−0.153	−0.155	**−0.225**	**−0.200**
Mon	0.019	−0.015	−0.086	−0.069	−0.116	−0.039	−0.147	−0.128	−0.166	**−0.195**
Rdw-Cv	0.136	−0.020	0.034	−0.136	0.055	0.001	−0.037	0.116	**0.222**	0.136
Rdw-Sd	**0.189**	0.059	0.127	−0.038	−0.015	0.066	0.094	**0.278**	**0.233**	**0.257**
Total Protein	−0.229	−0.113	**−0.299**	−0.121	−0.148	−0.193	−0.174	−0.157	**−0.320**	−0.256
Albumin	−0.094	0.027	−0.117	0.103	0.020	−0.010	−0.053	−0.045	**−0.194**	−0.103
Cholesterol	−0.074	0.047	−0.062	−0.025	−0.076	−0.007	0.003	−0.005	−0.088	−0.047
Tg	−0.043	−0.090	−0.113	−0.137	−0.187	−0.121	**−0.278**	−0.115	**−0.117**	−0.190

Abbreviations given in the Table: WBC: white blood cell; HGB: hemoglobin; MCW: mean corpuscular volume; MCH: mean corpuscular hemoglobin; MCHC: mean erythrocyte hemoglobin concentration; MPV: mean platelet volume; PCT: percent blood thrombus; PDW: platelet distribution width; NEU: neutrophil; LYM: lymphocyte; MON: monocyte; RDW-CV: red blood cell distribution width; RDW-SD: red blood cell distribution width—standard deviation value; TG: triglyceride; BWAS-1: Barratt–Warren Aggression Scale—physical aggression; BWAS-2: Barratt–Warren Aggression Scale—verbal aggression; BWAS-3: Barratt–Warren Aggression Scale—anger; BWAS-4: Barratt–Warren Aggression Scale—hostility; BWAS-5: Barratt–Warren Aggression Scale—indirect aggression; BWAS-6: Barratt–Warren Aggression Scale—total score. BIS-11-1: Barratt Impulsiveness Scale—attentional impulsiveness; BIS-11-2: Barratt Impulsiveness Scale—motor impulsiveness; BIS-11-3: Barratt Impulsiveness Scale—non-planning impulsiveness; BIS-11-4: Barratt Impulsiveness Scale—total impulsiveness score. Spearman’s Correlation Analysis was used in calculations. The r values are given in the Table; values in bold are calculated as *p* < 0.05.

**Table 5:** Logistic regression analysis results.

	B	S.E.	*p*	OR	%95 GA	
					Min	Max
Marital Status	−1.105	1.108	0.318	0.331	0.038	2.904
Social Security	0.509	0.813	0.531	1.664	0.338	8.191
Are Parents Together?	3.392	0.835	**0.000**	29.711	5.789	152.494
Prison Entrance Before	2.903	0.798	**0.000**	18.234	3.813	87.192
Tattoo/Tattoo	1.206	1.315	0.359	3.340	0.254	43.976
PANSS-Positive	−0.032	0.337	0.924	0.968	0.501	1.873
PANSS-Negative	−0.051	0.317	0.872	0.950	0.511	1.767
PANSS General Functionality	−0.120	0.348	0.730	0.887	0.449	1.752
PANNS Total Score	0.078	0.334	0.815	1.081	0.561	2.082
BWAS-Physical Aggression	0.002	0.232	0.992	1.002	0.636	1.580
BWAS-Total Score	−0.018	0.074	0.805	0.982	0.849	1.136
BIS-11 Unable To Plan	0.069	0.140	0.623	1.071	0.814	1.410
Mch	3.560	1.563	**0.023**	35.172	1.642	753.284
Mchc	−1.868	1.434	0.193	0.154	0.009	2.564
Mpv	−1.722	1.172	0.142	0.179	0.018	1.777
Pdw	0.033	0.046	0.481	1.033	0.943	1.132
Eos	−0.323	1.012	0.750	0.724	0.100	5.261
Rdw-Cv	7.169	2.612	**0.006**	1298.157	7.764	217,066.441
Rdw-Sd	−2.273	1.059	**0.032**	0.103	0.013	0.822
Creatinine	−10.872	5.640	0.054	00.000	0.000	1.201
Albumin	−0.355	0.257	0.168	0.702	0.424	1.161

## Data Availability

The data are not publicly available.
